# Unforeseen COVID-19 on Oncologic Bone Scan with SPECT/CT in a High Prevalence Area

**DOI:** 10.4274/mirt.galenos.2020.76059

**Published:** 2020-10-19

**Authors:** Sana Munir Gill, Aamna Hassan, Humayun Bashir

**Affiliations:** 1 Shaukat Khanum Memorial Cancer Hospital and Research Center, Department of Nuclear Medicine, Lahore, Pakistan

**Keywords:** Bone scan, single photon emission computed tomography/computed tomography, SARS-CoV-2, COVID-19

## Abstract

A 65-year-old woman with known diabetes and hypertension underwent a technetium methylene diphosphonate (Tc-99m MDP) bone scan with single photon emission computed tomography/computed tomography (SPECT/CT) for shoulder pain. She was initially treated for breast cancer and later for hepatocellular carcinoma. SPECT/CT showed MDP nonavid and scattered pulmonary ground-glass opacities bilaterally along with rounded nodular densities. Another 56-year-old patient who was newly diagnosed with right breast invasive ductal carcinoma underwent a bone scan with SPECT/CT, which revealed bilateral pulmonary infiltrates. Both patients later tested positive for Coronavirus Disease-2019 (COVID-19). Therefore, nuclear physicians should be watchful of findings related to COVID-19 on SPECT/CT thorax as this is becoming the new normal.

## Abstract (tr)

Altmış beş yaşında, diyabetik ve hipertansif olduğu bilinen kadın hastaya omuz ağrısı için tek foton emisyonlu bilgisayarlı tomografi/bilgisayarlı tomografi (SPECT/BT) ile teknesyum metilen difosfonat (Tc-99m MDP) kemik taraması yapıldı. Hasta başlangıçta meme kanseri ve daha sonra hepatosellüler karsinom tedavisi görmüştü. SPECT/BT; MDP tutmayan, iki yanlı, dağınık pulmoner buzlu cam opasiteleri ve yuvarlak nodüler dansiteler gösterdi. Başka bir 56 yaşında yeni teşhis edilmiş sağ memede invaziv duktal karsinomu olan hastada, SPECT/BT ile kemik taraması bilateral pulmoner infiltrasyonları ortaya çıkardı. Bu hastaların her ikisinde de daha sonra Koronavirüs Hastalığı-2019 (COVID-19) testi pozitif saptandı. Bu nedenle, nükleer tıp hekimleri SPECT/BT’de COVID-19’un toraks bulgularına dikkat etmelidir çünkü bu onlar için yeni normal haline gelmiştir.

## Figures and Tables

**Figure 1A, B, C, D, E, F, G:**
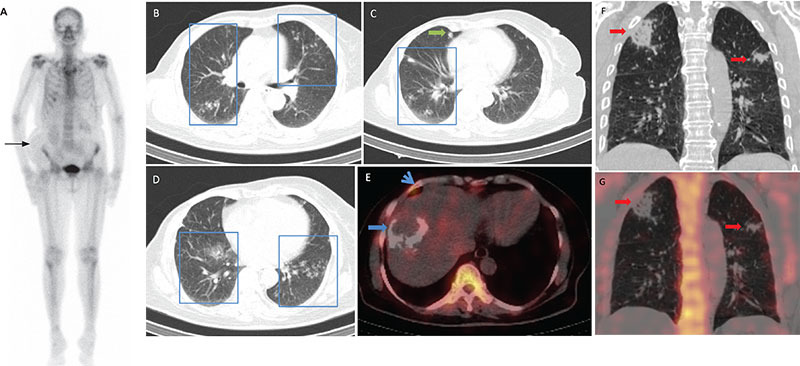
No evidence of osteoblastic metastases was seen on the whole-body planar bone scintigraphy anterior image (A) of a 65-year-old woman who had previously undergone modified radical mastectomy and chemotherapy for breast cancer. Spigelian hernia was noted on the right side of the abdomen (black arrow). Some heterogeneity was visible in the sacroiliac joints, which is likely to be degenerative in nature. No abnormal uptake was observed in the right shoulder. The uptake in the right anterior costochondral junction corresponded to an arthritic change on single photon emission computed tomography/computed tomography (SPECT/CT) (F: blue arrow head). Axial images of low-dose CT of correlative SPECT/CT (B, C, D) showed scattered and predominantly peripheral ground-glass opacities (GGO) in both lungs (blue box) and a couple of scattered rounded nodular densities (green arrow). Fused SPECT/CT image (E) revealed a calcified site of prior transarterial chemoembolization which was performed for treating second primary hepatocellular carcinoma (blue arrow). In the coronal CT (F) and fuzed SPECT/CT (G) images of another 56-year-old patient, pulmonary infiltrates were observed in the bilateral upper lung lobes (red arrows); the largest consolidative area was in the right upper lobe. The scan was acquired as part of the staging workup related to breast carcinoma. Detailed history of the patients revealed the absence of coughing, shortness of breath, fever, myalgia, diarrhea, or vomiting. Besides, there was no history of contact with a suspected or confirmed case of severe acute respiratory syndrome-coronavirus-2 (SARS-CoV-2). Objectively, they were afebrile and normotensive; oxygen saturation was 98% on room air. In view of the current Coronavirus Disease-2019 (COVID-19) pandemic, suspicion of the disease was raised despite the negative history owing to preexisting comorbidities, GGO, and pulmonary infiltrates on SPECT/CT. One study established that up to 97% of the confirmed COVID-19 patients exhibited GGO on CT irrespective of the severity of the disease (1). Therefore, the patients’ nasopharyngeal swabs were subjected to reverse transcriptase-polymerase chain reaction (RT-PCR), which turned out to be positive for SARS-CoV-2 infection 24 hours later. Hence, the patients were advised self-isolation, and they continued to be asymptomatic. COVID-19 is a rapidly emerging disease with over 9,373,719 patients affected worldwide (2) and continues to be a public health challenge. Ever since the initial cases were reported in December 2019 (3), there have been variable presentations with poor prognosis in certain age groups. Thus, a high index of suspicion is required for its diagnosis, especially in asymptomatic patients. To curtail the spread of the pandemic, early recognition and isolation of the affected people are of paramount importance. In such a scenario, hybrid imaging is playing a central role in flagging potential COVID-19 patients with incidental lung findings on positron emission tomography/CT (PET/CT) and SPECT/CT. The benefit of PET/CT has been documented in literature (4,5). However, there have been only a few reported incidental cases on SPECT/CT (6). In the constantly changing situation in our country that has a current tally of 188,925 (7) confirmed COVID-19 cases, most of the patients are asymptomatic. Hence, there is a high chance of these patients getting missed out on hybrid imaging performed for other unrelated causes. Therefore, nuclear medicine physicians should be aware of the COVID-19 related findings on thorax CT and inform the referring clinician if suspicious GGO are detected (8). Although RT-PCR is currently considered the gold standard for the diagnosis of the disease, some studies have highlighted the importance of combining the results with imaging, laboratory, and clinical findings. This approach is essential since many patients present with either nil or variable symptoms and might have a negative RT-PCR test (9)
